# Phase Engineering of TiO_2_/MXene Heterostructure Nanosheets for Enhanced Photocatalysis

**DOI:** 10.3390/ma19122663

**Published:** 2026-06-20

**Authors:** Yuntao Huang, Zibo Chen, Zhenyu Gong, Zhihong Dai, Cheng Chen, Daping He

**Affiliations:** 1 Sanya Science and Education Innovation Park, Wuhan University of Technology, Sanya 572000, China; 2 School of Materials Science and Engineering, Wuhan University of Technology, Wuhan 430070, China; 3 State Key Laboratory of Advanced Technology for Materials Synthesis and Processing, Wuhan University of Technology, Wuhan 430070, China; 4 Hubei Engineering Research Center of RF-Microwave Technology and Application, School of Physics and Mechanics, Wuhan University of Technology, Wuhan 430070, China

**Keywords:** phase engineering, heterostructure, photocatalysis

## Abstract

TiO_2_-based heterostructures have attracted considerable attention in photocatalytic pollutant degradation owing to their enhanced photoresponse and improved charge separation. The phase structure of TiO_2_ strongly affects its band structure and interfacial charge-transfer behavior, making phase structure control critical for optimizing photocatalytic performance. However, due to the small difference in free energy among TiO_2_ phase structure and the strong dependence of TiO_2_ nucleation and growth on the local reaction environment, it remains challenging to precisely control the phase structure of TiO_2_ in the TiO_2_-based heterostructure nanomaterials. Herein, we achieved the phase engineering of TiO_2_/MXene heterostructure nanomaterials through a solvent-regulation strategy. Specifically, by regulating the acetonitrile/water ratio in the hydrothermal solvent, TiO_2_ with distinct phase structures was in situ grown on hydrothermally treated MXene nanosheets, resulting in two representative TiO_2_/MXene heterostructure nanosheets: anatase TiO_2_/MXene and rutile TiO_2_/MXene. Acetonitrile likely acted as a surface-adsorbing agent during TiO_2_ formation, stabilizing the anatase phase and promoting the preferential formation of anatase TiO_2_. Benefiting from the optimized heterostructure, TiO_2_/MXene heterostructure nanosheets promoted the generation of singlet oxygen (^1^O_2_), leading to enhanced photocatalytic degradation.

## 1. Introduction

TiO_2_ has garnered attention in the field of photocatalytic pollutant degradation due to its excellent photostability, chemical stability, and environmental friendliness [1,2,3,4]. However, the inherent inability of TiO_2_ to absorb visible light and its rapid electron-hole recombination significantly limit its photocatalytic efficiency. In recent years, researchers have explored various strategies, including crystal morphology regulation, surface engineering, elemental doping, and heterostructure construction, to address the inherent limitations of TiO_2_ [5,6,7,8,9]. Among these strategies, coupling TiO_2_ with other materials to construct heterostructures can modulate its band structure, thereby enhancing its photoresponse and charge separation efficiency [10,11]. The TiO_2_/MXene heterostructure is a typical example, in which the in situ growth of TiO_2_ on MXene under hydrothermal conditions can improve catalytic activity [12].

As is known, TiO_2_ typically exists in a multiple phase structure, such as an anatase and rutile structure, leading it to possess different band structures, surface atomic arrangements and charge transfer characteristics, which directly influence light absorption, carrier separation, and surface redox reactions [13,14,15,16,17]. Therefore, precisely controlling the phase structure in TiO_2_-based heterostructures is crucial for optimizing their photocatalytic performance. However, due to the relatively small differences in free energy between TiO_2_ polymorphs and the high sensitivity of TiO_2_ nucleation and growth kinetics to the local reaction environment, solvent composition, and interfacial interactions, TiO_2_ often forms mixed crystalline phases during the formation of TiO_2_-based heterostructures. Therefore, achieving precise control over the phase structure of TiO_2_ in TiO_2_-based heterostructure nanomaterials remains a challenge.

Herein, we achieved the phase structure control of TiO_2_/MXene heterostructure nanosheets through a solvent-regulation strategy. Specifically, two types of TiO_2_/MXene heterostructure nanosheets with different TiO_2_ phase structures were fabricated, of which one was anatase TiO_2_/MXene (A-TiO_2_/MXene) and the other was rutile TiO_2_/MXene (R-TiO_2_/MXene). The ratio of acetonitrile (ACN) to water in the hydrothermal solvent modulates the environment during TiO_2_ growth. ACN may act as a surface adsorbent, stabilizing the anatase phase and thus preventing the TiO_2_ phase transition.

## 2. Materials and Methods

### 2.1. Synthesis of Ti_3_C_2_T_x_ MXenes

Ti_3_AlC_2_ powders (10 g) were etched in LiF (16 g)/HCl (9 M, 200 mL) at 50 °C for 36 h. The product was washed via centrifugation until the supernatant became transparent. The supernatant obtained after centrifugation was collected to yield a dispersion of Ti_3_C_2_T_x_ nanosheets.

### 2.2. Synthesis of TiO_2_/MXene Heterostructure Nanosheets

Ti_3_C_2_T_x_ MXene (35 mg) was dispersed in 35 mL of ACN/H_2_O mixed solvents with different ACN volume fractions of 0, 25, 50, 75, and 100 vol% and ultrasonicated for 20 min to obtain homogeneous dispersions. The pH of the MXene dispersions was 6.7. The mixture was then transferred to a 50 mL Teflon-lined stainless-steel autoclave, heated to 120 °C, and maintained for 24 h, followed by natural cooling to room temperature. The obtained samples were washed with deionized water and collected by centrifugation at 10,000 rpm for 5 min. This washing and centrifugation process was repeated three times. The final products were then dried in a vacuum oven at 50 °C for 6 h and collected. Anatase TiO_2_ grown in situ on the MXene surface in pure ACN was obtained and designated as A-TiO_2_/MXene nanosheets. Rutile TiO_2_ grown in situ on the MXene surface in pure water was obtained and designated as R-TiO_2_/MXene nanosheets.

### 2.3. Photocatalytic Degradation Performance

A total of 5 mg of the photocatalyst was dispersed in 50 mL of RhB solution (50 ppm). The suspension was stirred continuously in the dark for 30 min to establish adsorption–desorption equilibrium. Subsequently, the suspension was subjected to full-spectrum irradiation (λ > 300 nm) using a 300 W xenon lamp (Beijing Perfectlight). The irradiation was performed with the light source positioned 10 cm away from the suspension, an irradiation intensity of 100 mW/cm^2^, and the reaction temperature maintained at 20 °C. During the reaction, the suspension was magnetically stirred at 400 rpm to ensure uniform dispersion of the catalyst. A 2 mL aliquot of the reaction mixture was sampled every 10 min and analyzed after centrifugation at 10,000 rpm for 5 min. RhB concentration was estimated by UV-vis spectroscopy (UV-3600i Plus). For comparison, phenol solution (20 ppm) was used as a non-dye organic pollutant under the same photocatalytic conditions. The initial pH values of the RhB and phenol solutions were not adjusted, and the solutions were used at their intrinsic pH values corresponding to the specified concentrations. Total organic carbon (TOC) analysis was further performed to evaluate the mineralization degree during RhB degradation. Electron paramagnetic resonance (EPR) spectra were obtained on an EPR spectrometer (MEX-nano, Bruker, Coventry, UK) with a modulation frequency of 100 kHz and a microwave power of 25 mW.

### 2.4. Photoelectrochemical Measurements

The photoelectrochemical tests were conducted with a three-electrode electrochemical workstation (Chenhua CHI660, Shanghai Chenhua Instrument Co., Ltd., Shanghai, China) in a conventional three-electrode mode. An F–doped tin oxide (FTO) glass coated with the tested sample, a platinum sheet and Ag/AgCl were used as working, counter and reference electrodes, respectively. The working electrode was prepared as follows: 5 mg of sample was uniformly dispersed in the mixture of deionized water (750 μL), nafion (50 μL; 5%) and ethanol (250 μL) by sonication for 1 h. Then, 30 µL of the suspension was dip-coated onto an FTO glass (1 × 1 cm^2^), and the covered FTO was vacuum dried at 50 °C for 3 h. A 300 W xenon lamp and an aqueous solution of sodium sulfate (0.5 M) were used as an irradiation source and an electrolyte, respectively.

## 3. Results and Discussion

**Figure 1:**
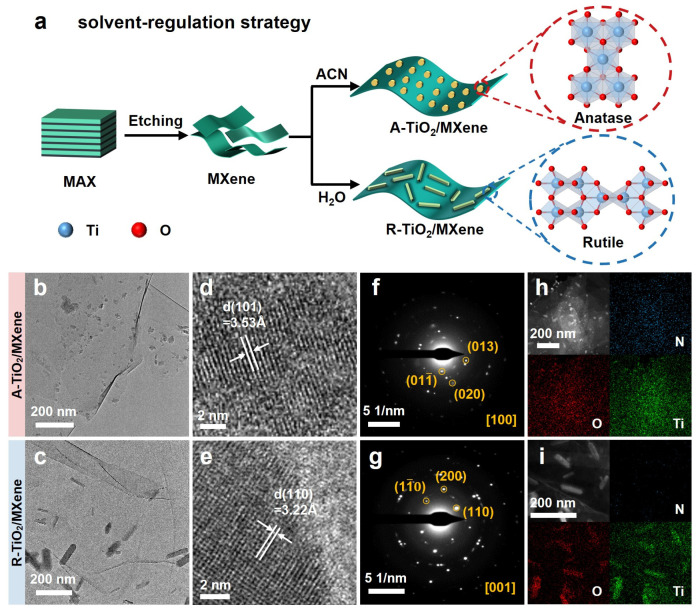
(**a**) Synthetic schematic, (**b**,**c**) TEM images, (**d**,**e**) HRTEM images, (**f**,**g**) SAED patterns, and (**h**,**i**) EDS elemental mapping images of A-TiO_2_/MXene nanosheets and R-TiO_2_/MXene nanosheets.

The phase structure of TiO_2_ on MXene nanosheets was regulated via a solvent-regulation strategy. As shown in Figure 1a, A-TiO_2_/MXene nanosheets and R-TiO_2_/MXene nanosheets were in situ hydrothermally synthesized in ACN and water, respectively. To investigate the structures of the A-TiO_2_/MXene and R-TiO_2_/MXene nanosheets, transmission electron microscopy (TEM) images, scanning electron microscopy (SEM) images, high-resolution transmission electron microscopy (HRTEM) images and selected area electron diffraction (SAED) patterns were employed. As shown in Figure 1b,c, Figures S1 and S2, the average particle size of the anatase TiO_2_ nanoparticles on the A-TiO_2_/MXene nanosheets is approximately 15 nm. The particle size of the rutile TiO_2_ rod-shaped particles on the R-TiO_2_/MXene nanosheets is approximately 50 nm. As shown in Figure 1d, the lattice spacing of the A-TiO_2_/MXene nanosheets is 0.353 nm, which is attributed to the (101) crystal plane of the anatase TiO_2_ [18]. Meanwhile, the SAED pattern of the A-TiO_2_/MXene nanosheets exhibited diffraction spots of a body-centered tetragonal structure (Figure 1f). As shown in the HRTEM image of the R-TiO_2_/MXene nanosheets (Figure 1e), the lattice spacing of the R-TiO_2_/MXene nanosheets is 0.322 nm, which is attributed to the (110) plane of the rutile TiO_2_ [19]. Simultaneously, the SAED pattern of the R-TiO_2_/MXene nanosheets exhibited diffraction spots of a simple tetragonal structure (Figure 1g). Moreover, the energy dispersive spectroscopy (EDS) mapping (Figure 1h,i) results confirmed a uniform dispersion for Ti and O elements on the surfaces of the A-TiO_2_/MXene nanosheets and R-TiO_2_/MXene nanosheets. Notably, N was detected on the A-TiO_2_/MXene nanosheets, likely originating from the ACN solvent, suggesting ACN was adsorbed on the TiO_2_ surface.

**Figure 2:**
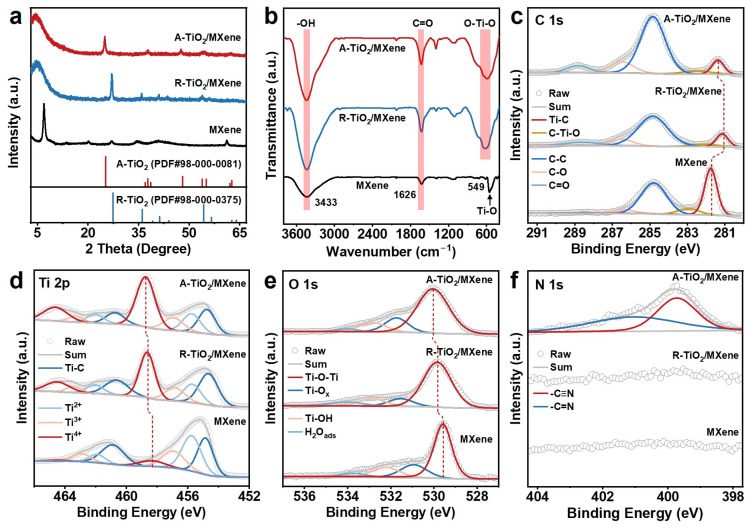
(**a**) XRD patterns, (**b**) FTIR spectra, and XPS high-resolution spectrum of (**c**) C 1s, (**d**) Ti 2p, (**e**) O 1s, and (**f**) N 1s of MXene, A-TiO_2_/MXene and R-TiO_2_/MXene nanosheets.

To further investigate the phase structure between the A-TiO_2_/MXene and R-TiO_2_/MXene nanosheets, an X-ray diffraction (XRD) pattern was obtained. As shown in the XRD patterns (Figure 2a), the A-TiO_2_/MXene nanosheets exhibited a characteristic peak at 25.3°, corresponding to the (101) plane of anatase TiO_2_. Meanwhile, the R-TiO_2_/MXene nanosheets displayed a peak at 27.4°, corresponding to the (110) plane of rutile TiO_2_ [20]. The XRD results are consistent with the HRTEM images and SAED results, suggesting the phase regulation of TiO_2_ in the TiO_2_/MXene nanosheets is successfully achieved. To elucidate the chemical bonding of each component within TiO_2_/MXene heterostructure nanosheets, various spectroscopic analyses were employed. As shown in the Fourier transform infrared spectroscopy (FTIR) spectra (Figure 2b), the hydroxyl (-OH) and carbonyl (C=O) stretching vibration peaks of the MXene were located at 3438 cm^−1^ and 1630 cm^−1^ [21]. The peaks at 549 cm^−1^ of the MXene are assigned as the Ti-O deformation vibration between the terminated -OH and the titanium atom on the surface of the MXene. In addition, the broad peak around 600 cm^−1^ of the A-TiO_2_/MXene and R-TiO_2_/MXene can be attributed to the O-Ti-O lattice stretching vibration of TiO_2_ [22], indicating that TiO_2_ was formed on the MXene. X-ray photoelectron spectroscopy (XPS) was utilized to explore the surface chemical state and charge transfer. In the C 1s XPS spectra (Figure 2c), the Ti-C peak of pristine MXene located at 281.7 eV shifts to lower binding energies of 281.3 and 281.1 eV for the A-TiO_2_/MXene and R-TiO_2_/MXene nanosheets, respectively. Due to the Ti-C signal originating from the MXene framework, the negative shift in Ti-C peak indicates increased electron density on the MXene after coupling with TiO_2_. In the Ti 2p spectra (Figure 2d), the enhanced Ti^4+^ peaks in the A-TiO_2_/MXene and R-TiO_2_/MXene nanosheets confirm the formation of TiO_2_ on the MXene [23]. Compared with the MXene (458.3 eV), the Ti^4+^ peaks of the A-TiO_2_/MXene nanosheets and R-TiO_2_/MXene nanosheets shift to higher binding energies of 458.6 and 458.7 eV, respectively, suggesting that the Ti^4+^ centers in the TiO_2_ become relatively electron-deficient after interfacial coupling with the MXene. In the O 1s spectra (Figure 2e), the Ti-O-Ti-related component shifts from 529.6 eV in the MXene to 530.0 and 529.8 eV in the A-TiO_2_/MXene nanosheets and R-TiO_2_/MXene nanosheets, respectively, indicating that the local chemical environment of Ti-O bonds is modified after TiO_2_ growth on the MXene. These XPS results demonstrate the existence of interfacial electronic interactions and charge redistribution between TiO_2_ and MXene nanosheets [24]. In the N 1s spectrum (Figure 2f), the peaks of the A-TiO_2_/MXene nanosheets at 399.7 and 401.0 eV are assigned to the cyano groups (–C≡N) and imine groups (–C=N) of ACN, respectively [25], indicating the adsorption or retention of ACN-derived species on the A-TiO_2_/MXene nanosheet surface, which agrees with EDS elemental mapping data. According to the comprehensive results of SEM, TEM, XRD, FTIR and XPS characterization, the A-TiO_2_/MXene nanosheets and R-TiO_2_/MXene nanosheets were obtained by modulating the solvent system during the hydrothermal reaction of the MXene. Specifically, an ACN-based system promotes the crystallization of A-TiO_2_, whereas a pure aqueous system induces the crystallization of R-TiO_2_. It is convincing to conclude that ACN may act as a surface-adsorbing agent during TiO_2_ growth, stabilizing the anatase phase and thereby promoting the preferential formation of anatase TiO_2_.

**Figure 3:**
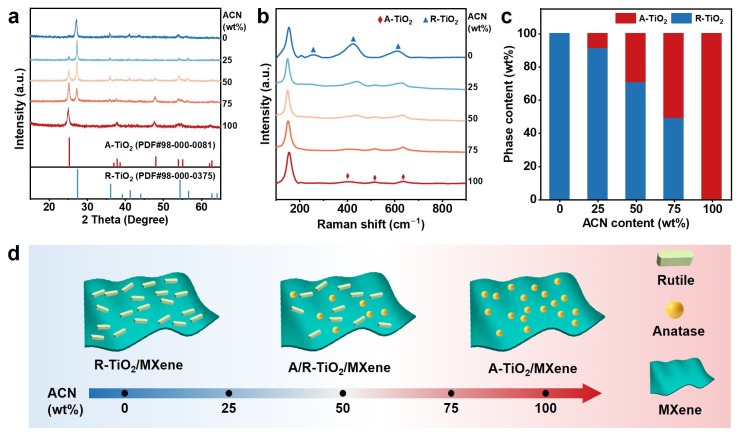
(**a**) XRD patterns, (**b**) Raman spectra, and (**c**) phase content of TiO_2_/MXene nanosheets at different ACN content. (**d**) Schematics for the solvent-regulation strategy.

To investigate the effect of the solvent composition on the formation of TiO_2_ phase structure on MXene nanosheets, the proportion of ACN in the hydrothermal solvent was regulated and the corresponding XRD and Raman characterization were employed. As the ACN content increased, the XRD patterns of the TiO_2_/MXene nanosheets exhibited an increased intensity of the anatase (101) characteristic peak and a decreased intensity of the rutile (110) characteristic peak, indicating an increase in the anatase TiO_2_ content and a decrease in the rutile TiO_2_ content within the TiO_2_/MXene nanosheets (Figure 3a). Similarly, the Raman spectra (Figure 3b) of the TiO_2_/MXene nanosheets displayed peak variation trends of an increased intensity of the anatase characteristic peak, showing good consistency with the XRD results, suggesting that ACN promotes the formation of A-TiO_2_. To quantitatively analyze the relationship between the solvent composition and the crystalline phase composition of TiO_2_, Rietveld XRD analysis of the TiO_2_/MXene heterostructure nanosheets was performed. As shown in Figure 3c and Figure S3, as the ACN content in the solution increases to 0%, 25%, 50%, 75%, and 100%, the A-TiO_2_ content within the TiO_2_/MXene nanosheets reaches 0%, 8.4%, 29%, 50.7% and 100%, respectively. Based on the XRD and Raman results, the solvent-regulation mechanism is schematically illustrated in Figure 3d. By regulating the ACN/water ratio in the hydrothermal solvent, the TiO_2_ phase structure could be effectively tailored from rutile-dominated to anatase-dominated structures within TiO_2_/MXene heterostructure nanosheets.

**Figure 4:**
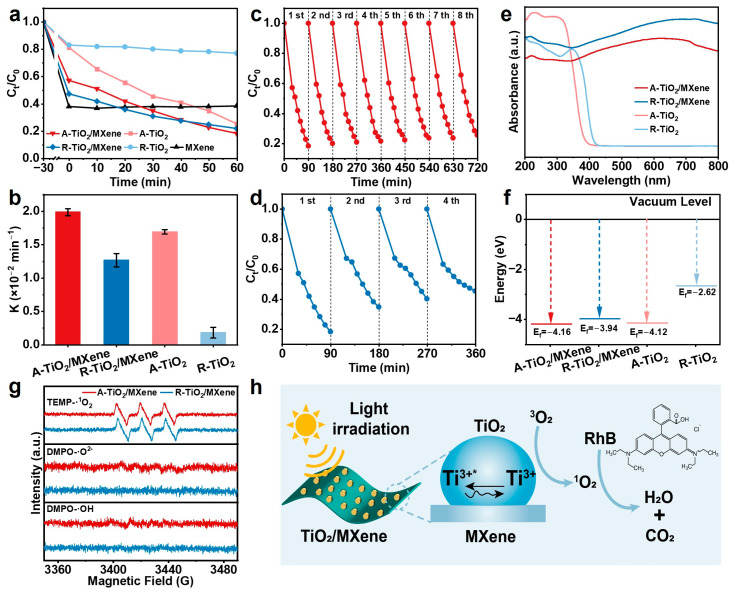
(**a**) RhB concentration variation curves and (**b**) photodegradation rate constants of A-TiO_2_/MXene nanosheets, R-TiO_2_/MXene nanosheets, A-TiO_2_, R-TiO_2_ and MXene under full-spectrum irradiation. (**c**,**d**) Cyclic degradation curves of A-TiO_2_/MXene nanosheets and R-TiO_2_/MXene nanosheets. (**e**) The UV-Vis DRS spectra of A-TiO_2_/MXene nanosheets, R-TiO_2_/MXene nanosheets, A-TiO_2_ and R-TiO_2_. (**f**) Fermi level of A-TiO_2_/MXene nanosheets, R-TiO_2_/MXene nanosheets, A-TiO_2_ and R-TiO_2_ (vs. Vacuum Level). (**g**) EPR spectra of TEMP−^1^O_2_ adducts (in water solution), DMPO-•OH adducts (in water solution) and DMPO-•O_2_^−^ adducts (in methanol solution) over A-TiO_2_/MXene nanosheets and R-TiO_2_/MXene nanosheets under irradiation for 3 min. (**h**) Schematic diagram of the photocatalytic mechanism of the TiO_2_/MXene nanosheets. The asterisk (*) denotes an electronically excited state.

The photocatalytic activities of the A-TiO_2_/MXene and R-TiO_2_/MXene nanosheets were evaluated using RhB as the target pollutant under full-spectrum irradiation. For comparison, the photocatalytic activities of commercial anatase TiO_2_ nanoparticles (A-TiO_2_), commercial rutile TiO_2_ nanoparticles (R-TiO_2_), and MXene were also tested. As shown in Figure 4a, MXene exhibited no significant catalytic activity. In contrast, the A-TiO_2_/MXene nanosheets achieved a degradation efficiency of 81.8%, while the R-TiO_2_/MXene nanosheets reached 78.2%, both surpassing the performance of A-TiO_2_ (74.7%) and R-TiO_2_ (22.9%). To further distinguish dye decolorization from organic carbon removal, total organic carbon (TOC) analysis was performed after RhB photocatalytic degradation. As shown in Figure S4, the TOC removal efficiencies of the A-TiO_2_/MXene nanosheets, R-TiO_2_/MXene nanosheets, A-TiO_2_ and R-TiO_2_ were 57.9%, 48.2%, 53.7% and 11.6%, respectively. The TOC removal efficiencies were lower than the RhB removal efficiencies obtained from UV-vis measurements, indicating that the decrease in the RhB absorption peak mainly reflects decolorization and degradation of the chromophoric structure [26]. Furthermore, the A-TiO_2_/MXene nanosheets exhibited the highest TOC removal efficiency among all the tested samples, suggesting the superior photocatalytic activity of A-TiO_2_/MXene nanosheets towards RhB degradation.

To evaluate the kinetic data for the photocatalytic degradation, the reaction rate constant (k) was determined by fitting a pseudo-first-order kinetic model. As shown in Figure 4b, the photodegradation rate constant (k) of the A-TiO_2_/MXene nanosheets was 1.940 × 10^−2^ min^−1^, which was higher than those of the R-TiO_2_/MXene nanosheets (1.294 × 10^−2^ min^−1^) and A-TiO_2_ (1.726 × 10^−2^ min^−1^), indicating that the A-TiO_2_/MXene nanosheets exhibited faster RhB degradation kinetics. Compared to the R-TiO_2_/MXene nanosheets with lower degradation rate, the degradation rate of the A-TiO_2_/MXene nanosheets remained unchanged after eight cycles. Moreover, the XRD pattern and SEM image (Figure S5) of the A-TiO_2_/MXene nanosheets after cycling tests showed no obvious change in their structure, indicating that A-TiO_2_/MXene nanosheets possess good structural stability. Additionally, considering that RhB is a dye molecule and may undergo photosensitization under irradiation, phenol was also selected as a non-dye organic pollutant to further evaluate the photocatalytic degradation performance of TiO_2_/MXene nanosheets. As shown in Figure S6, the A-TiO_2_/MXene nanosheets exhibited the highest photocatalytic activity towards phenol degradation, with an apparent rate constant of 1.09 × 10^−2^ min^−1^, which was higher than those of the R-TiO_2_/MXene nanosheets (0.47 × 10^−2^), A-TiO_2_ (0.76 × 10^−2^) and R-TiO_2_ (0.11 × 10^−2^ min^−1^), respectively. The phenol photocatalytic degradation results reveal that the enhanced photocatalytic activity of A-TiO_2_/MXene nanosheets is also applicable to the degradation of non-dye organic pollutants.

Furthermore, to investigate the optical properties of the TiO_2_/MXene nanosheets, we employed ultraviolet–visible diffuse reflectance spectroscopy (DRS) (Figure 4e). Compared with A-TiO_2_ and R-TiO_2_, the A-TiO_2_/MXene and R-TiO_2_/MXene nanosheets showed significantly enhanced visible-light absorption. The enhanced visible-light absorption of the TiO_2_/MXene heterostructure nanosheets was mainly attributed to the introduction of MXene, which improved the light-harvesting capability of the TiO_2_/MXene heterostructure nanosheets [27]. To assess the charge transfer trend of the sample, ultraviolet photoelectron spectroscopy (UPS) was used to determine its work function. As shown in Figure S7, the work functions of the A-TiO_2_/MXene nanosheets, R-TiO_2_/MXene nanosheets, A-TiO_2_, and R-TiO_2_ are determined to be 4.16 eV, 3.94 eV, 4.12 eV, and 2.62 eV, respectively. Based on the UPS measurement result, the Fermi level positions are shown in Figure 4f. The Fermi level of the A-TiO_2_/MXene nanosheets is located at −4.16 eV, which is deeper than those of the R-TiO_2_/MXene nanosheets, A-TiO_2_ and R-TiO_2_. The deeper Fermi level indicates that the A-TiO_2_/MXene nanosheets had a larger work function and stronger electron-accepting tendency, which may facilitate the extraction and transfer of photogenerated electrons. As shown in Figure S8, the A-TiO_2_/MXene nanosheets exhibited the higher photocurrent response among the tested samples, indicating more efficient generation, separation and transport of photogenerated charge carriers, which is consistent with the UPS analysis of the enhanced interfacial charge-transfer behavior in the A-TiO_2_/MXene nanosheets.

To elucidate the reactive oxygen species (ROS) involved in the photocatalytic reaction, electron paramagnetic resonance (EPR) spectroscopy was performed using 2,2,6,6-tetramethylpiperidine (TEMP) and 5,5-dimethyl-1-pyrroline N-oxide (DMPO) as trapping agents, respectively. As shown in Figure 4g, the A-TiO_2_/MXene nanosheets and R-TiO_2_/MXene nanosheets displayed characteristic triplet signals of TEMP−^1^O_2_ under light irradiation, confirming the generation of ^1^O_2_ during the photocatalytic degradation process [28]. Conversely, the characteristic signals of DMPO-•OH or DMPO-•O_2_^−^ were not observed, indicating that •OH and •O_2_^−^ are not the main reactive oxygen species. These results indicate that A-TiO_2_/MXene and R-TiO_2_/MXene nanosheets generate ^1^O_2_ during the photocatalytic degradation process via a long-range energy transfer mechanism [29]. Based on the EPR analyses, the photocatalytic degradation mechanisms of RhB by A-TiO_2_/MXene and R-TiO_2_/MXene nanosheets are schematically summarized. As shown in Figure 4h, the intimate TiO_2_/MXene nanosheets interface induces interfacial charge redistribution, which may facilitate the formation and stabilization of Ti^3+^-associated defect states at the interface or in the subsurface region of TiO_2_. Under illumination, they tend to act as energy donors, utilizing long-range energy transfer to excite surface-adsorbed ^3^O_2_ into ^1^O_2_.

## 4. Conclusions

In conclusion, we prepared TiO_2_/MXene nanosheets with tunable TiO_2_ phase structure using a solvation control strategy. XRD and TEM confirmed that anatase TiO_2_ and rutile TiO_2_ were successfully in situ grown on MXene nanosheets. Meanwhile, Raman and Rietveld XRD analyses confirmed that the TiO_2_ phase structure in TiO_2_/MXene heterostructure nanosheets can be controlled by adjusting the ACN/water ratio in the hydrothermal solvent. Benefiting from the efficient formation of singlet oxygen, the obtained A-TiO_2_/MXene heterostructure nanosheets effectively facilitated the photocatalytic degradation of RhB, exhibiting enhanced photocatalytic activity and stability. This work sheds light on the phase engineering of TiO_2_-based heterostructure nanomaterials for photocatalytic pollutant degradation.

## Data Availability

The original contributions presented in this study are included in the article/Supplementary Material. Further inquiries can be directed to the corresponding authors.

## Supplementary Materials

The following supporting information can be downloaded at: https://www.mdpi.com/article/10.3390/ma19122663/s1, Figure S1: (a) SEM image, (b) TEM image and (c) particle size distribution of A-TiO_2_/MXene nanosheets; Figure S2: (a) SEM images, (b) TEM images and (c) particle size distribution of R-TiO_2_/MXene nanosheets; Figure S3: Rietveld refinement of TiO_2_/MXene nanosheets synthesized with ACN content of (a) 100%, (b) 75%, (c) 50%, (d) 25%, and (e) 0%.; Figure S4: TOC removal efficiencies of A-TiO_2_/MXene nanosheets, R-TiO_2_/MXene nanosheets, A-TiO_2_ and R-TiO_2_; Figure S5: (a) XRD pattern and (b) SEM image of A-TiO_2_/MXene nanosheets after eight photocatalytic cycles; Figure S6: (a) Phenol concentration variation curves and (b) photodegradation rate constants of A-TiO_2_/MXene nanosheets, R-TiO_2_/MXene nanosheets, A-TiO_2_ and R-TiO_2_ under full-spectrum irradiation; Figure S7: UV photoelectron spectrum of (a) A-TiO_2_/MXene nanosheets, (b) R-TiO_2_/MXene nanosheets, (c) A-TiO_2_ and (d) R-TiO_2_ secondary electron cut-off; Figure S8: Photocurrent responses curves of A-TiO_2_/MXene nanosheets, R-TiO_2_/MXene nanosheets, A-TiO_2_ and R-TiO_2_.
