# Supplementary material for: Phase Engineering of TiO2/MXene Heterostructure Nanosheets for Enhanced Photocatalysis
Source: Materials (Basel). 2026 Jun 20;19(12):2663. doi: 10.3390/ma19122663 (PMC13302986; doi:10.3390/ma19122663)
Supplement: Supplementary file 1 [file materials-19-02663-s001.zip › materials-4351340-supplementary.pdf]

Supporting Information for

Phase Engineering of TiO_2/MXene Heterostructure Nanosheets for Enhanced Photocatalysis

Yuntao Huang [1,2,†], Zibo Chen [1,2,†], Zhenyu Gong [1,2], Zhihong Dai [1,2], Cheng Chen [1,3,*], Daping He [1,4,*]

[1]Sanya Science and Education Innovation Park of Wuhan University of Technology, Sanya 572000, China;

[2]School of Materials Science and Engineering, Wuhan University of Technology, Wuhan 430070, China;

[3] State Key Laboratory of Advanced Technology for Materials Synthesis and Processing, Wuhan University of Technology, Wuhan 430070, China;

[4] Hubei Engineering Research Center of RF-Microwave Technology and Application, School of Physics and Mechanics, Wuhan University of Technology, Wuhan 430070, China.

*Correspondence: chengchen@whut.edu.cn (C. C.); hedaping@whut.edu.cn (D. H.)

[†]Yuntao Huang and Zibo Chen are co-first authors. These authors contributed equally to this work.

Materials

Ti_3AlC_2 MAX powders were purchased from 11 Technology Co., Ltd. (Jilin, China). Concentrated hydrochloric acid (HCl), lithium fluoride (LiF), Phenol and Rhodamine B (RhB) were purchased from Shanghai Macklin Biochemical Technology Co., Ltd. (Shanghai, China). Anatase TiO_2, rutile TiO_2, anhydrous acetonitrile (ACN), methanol (MeOH), 2,2,6,6-tetramethylpiperidine (TEMP), and 5,5-dimethyl-1-pyrroline N-oxide (DMPO) were purchased from Shanghai Aladdin Biochemical Technology Co., Ltd. (Shanghai, China).

Characterizations

Scanning electron microscopy (SEM) images and energy-dispersive X-ray spectroscopy (EDS) spectra of samples were captured on Zeiss Cross Beam 350 (**Crossbeam 350, Carl Zeiss Microscopy GmbH, Oberkochen, Germany**). Transmission electron microscopy (TEM) images of samples were captured on a JEOL JEM-F200 (**JEM-F200, JEOL Ltd., Akishima, Tokyo, Japan**). X-ray diffraction (XRD) patterns of samples were conducted on Rigaku Miniflex 600 (**MiniFlex 600, Rigaku Corporation, Akishima, Tokyo, Japan**) with Cu Kα radiation (λ = 1.5406 Å). Fourier transform infrared (FTIR) spectra of samples were obtained on Thermo Scientific Nicolet 6700 (**Nicolet 6700, Thermo Fisher Scientific, Madison, WI, USA**). Raman spectra of samples were collected on Thermo Scientific DXR3 (**DXR3, Thermo Fisher Scientific, Waltham, MA, USA**) with a laser wavelength of 532 nm. X-ray photoelectron spectroscopy (XPS) spectrum of samples was measured by Thermo Scientific ESCALAB 250Xi (**ESCALAB 250Xi, Thermo Fisher Scientific, East Grinstead, UK**). The work function of the samples were measured using an ESCALAB 250Xi ultraviolet photoelectron spectrometer (**ESCALAB 250Xi, Thermo Fisher Scientific, East Grinstead, UK**).

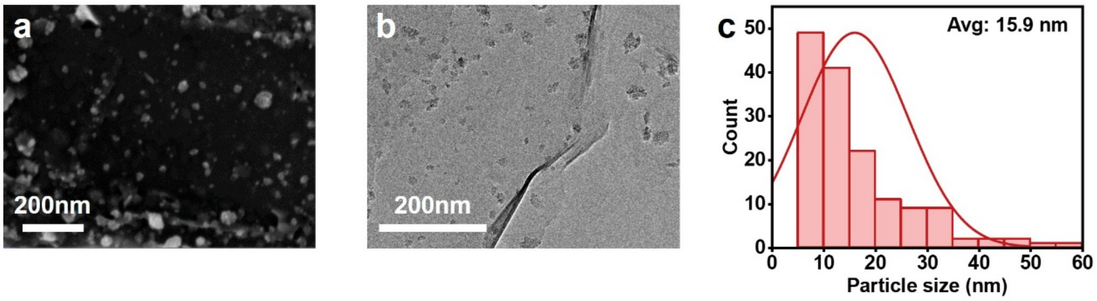

Figure S1. (a) SEM image, (b) TEM image and (c) particle size distribution of A-TiO_2/MXene nanosheets.

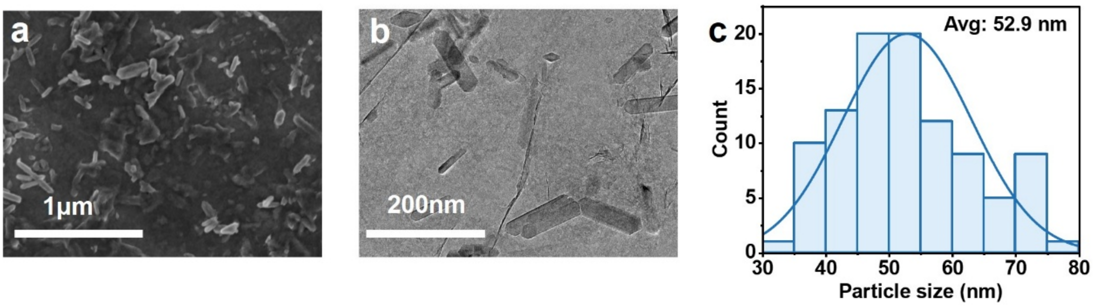

Figure S2. (a) SEM image, (b) TEM image and (c) particle size distribution of R-TiO_2/MXene nanosheets.

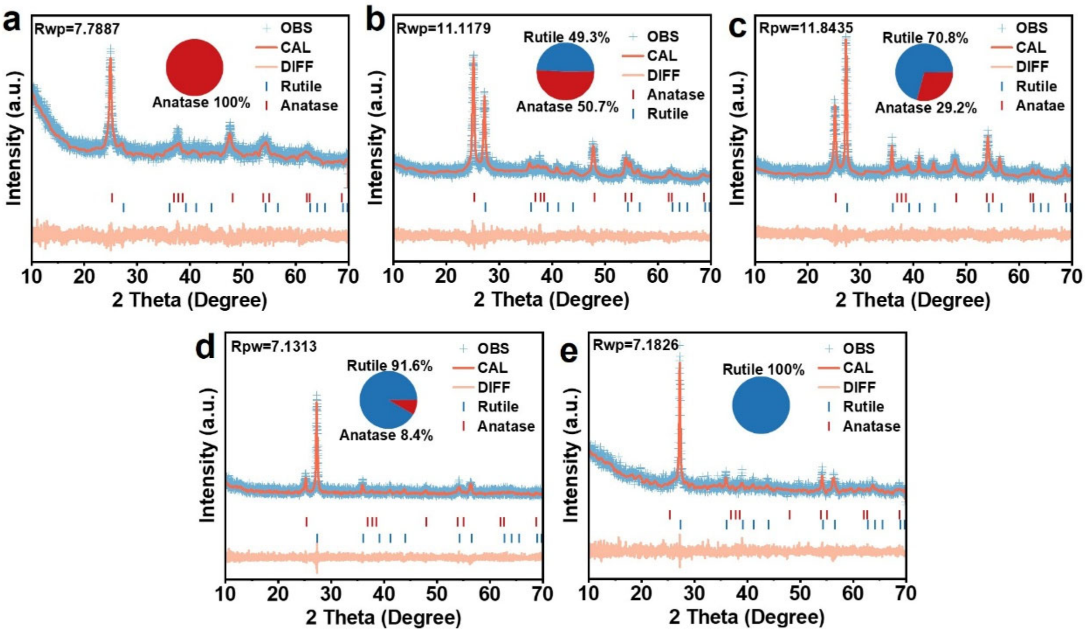

Figure S3. Rietveld refinement of TiO_2/MXene nanosheets synthesized with ACN content of (a) 100%, (b) 75%, (c) 50%, (d) 25%, and (e) 0%.

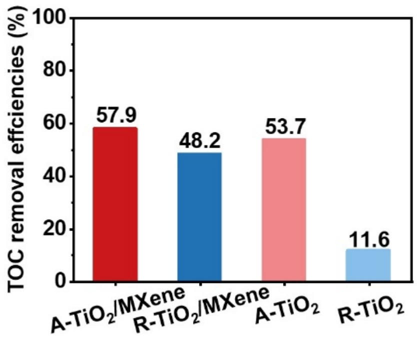

Figure S4. TOC removal efficiencies of A-TiO_2/MXene nanosheets, R-TiO_2/MXene nanosheets, A-TiO_2 and R-TiO_2.

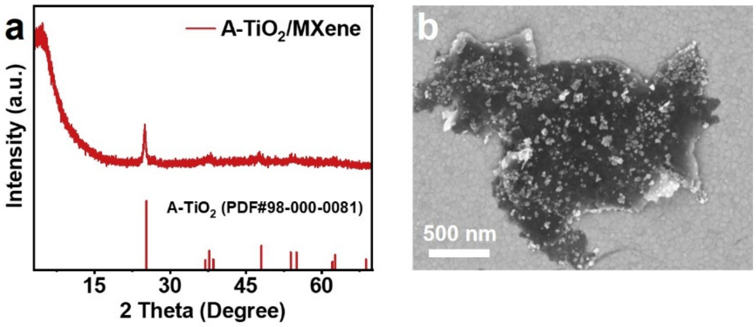

Figure S5. (a) XRD pattern and (b) SEM image of A-TiO_2/MXene nanosheets after eight photocatalytic cycles.

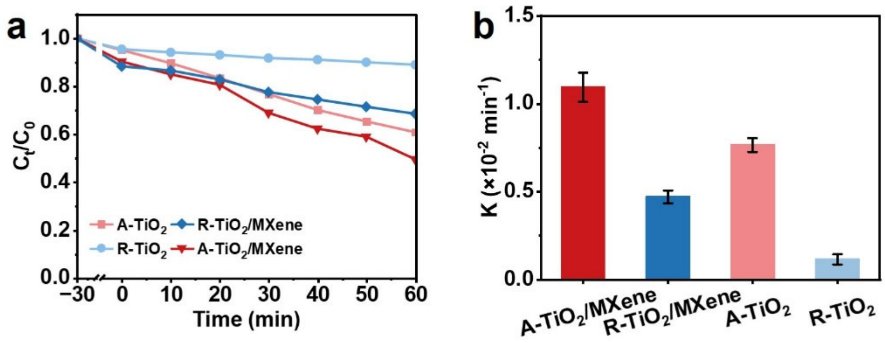

Figure S6. (a) Phenol concentration variation curves and (b) photodegradation rate constants of A-TiO_2/MXene nanosheets, R-TiO_2/MXene nanosheets, A-TiO_2 and R-TiO_2 under full-spectrum irradiation.

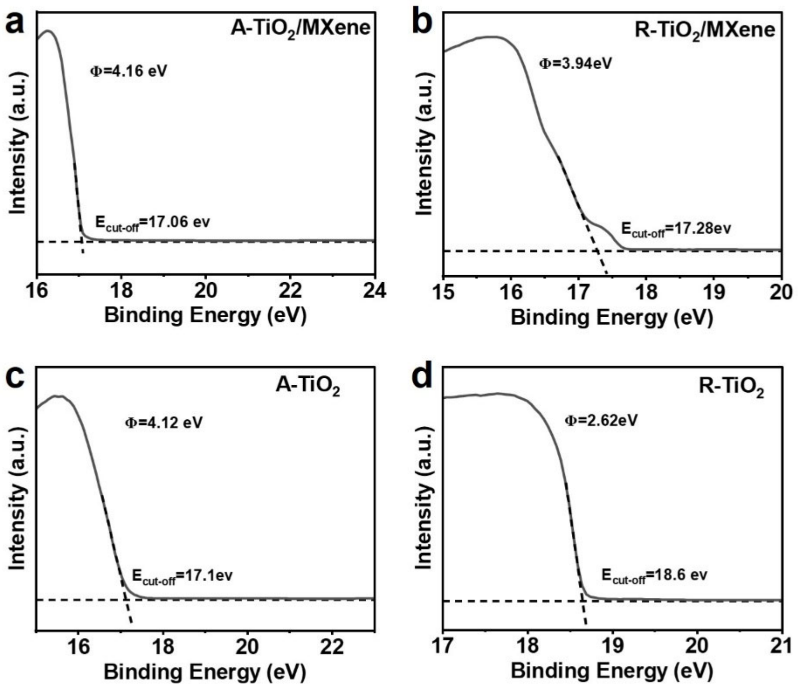

Figure S7. UV Photoelectron Spectrum of (a) A-TiO_2/MXene nanosheets, (b) R-TiO_2/MXene nanosheets, (c) A-TiO_2 and (d) R-TiO_2 secondary electron cut-off.

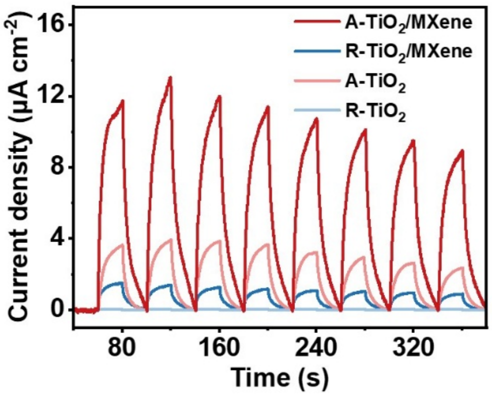

Figure S8. Photocurrent responses curves of A-TiO_2/MXene nanosheets, R-TiO_2/MXene nanosheets, A-TiO_2 and R-TiO_2.
